# Ultraconserved Elements in the *Olig2* Promoter

**DOI:** 10.1371/journal.pone.0003946

**Published:** 2008-12-16

**Authors:** Christina T. L. Chen, David I. Gottlieb, Barak A. Cohen

**Affiliations:** 1 Department of Genetics, Washington University in St. Louis School of Medicine, St. Louis, Missouri, United States of America; 2 Department of Anatomy and Neurobiology, Washington University in St. Louis School of Medicine, St. Louis, Missouri, United States of America; Indiana University, United States of America

## Abstract

**Background:**

Oligodendrocytes are specialized cells of the nervous system that produce the myelin sheaths surrounding the axons of neurons. Myelinating the axons increases the speed of nerve conduction and demyelination contributes to the pathology of neurodegenerative diseases such as multiple sclerosis. Oligodendrocyte differentiation is specified early in development by the expression of the basic-helix-loop-helix transcription factor *Olig2* in the ventral region of the neural tube. Understanding how *Olig2* expression is controlled is therefore essential for elucidating the mechanisms governing oligodendrocyte differentiation. A method is needed to identify potential regulatory sequences in the long stretches of adjacent non-coding DNA that flank *Olig2*.

**Methodology/Principal Findings:**

We identified ten potential regulatory regions upstream of *Olig2* based on a combination of bioinformatics metrics that included evolutionary conservation across multiple vertebrate genomes, the presence of potential transcription factor binding sites and the existence of ultraconserved elements. One of our computational predictions includes a region previously identified as the *Olig2* basal promoter, suggesting that our criterion represented characteristics of known regulatory regions. In this study, we tested one candidate regulatory region for its ability to modulate the *Olig2* basal promoter and found that it represses expression in undifferentiated embryonic stem cells.

**Conclusions/Significance:**

The regulatory region we identified modifies the expression regulated by the *Olig2* basal promoter in a manner consistent with our current understanding of *Olig2* expression during oligodendrocyte differentiation. Our results support a model in which constitutive activation of *Olig2* by its basal promoter is repressed in undifferentiated cells by upstream repressive elements until that repression is relieved during differentiation. We conclude that the potential regulatory elements presented in this study provide a good starting point for unraveling the cis-regulatory logic that governs *Olig2* expression. Future studies of the functionality of the potential regulatory elements we present will help reveal the interactions that govern *Olig2* expression during development.

## Introduction

Oligodendrocytes are glial cells that myelinate axons, forming the myelin sheaths in the central nervous system. These myelin sheaths enhance the saltatory conduction by insulating the axons and forming the nodes of Ranvier (reviewed in [1], [2]). Damage to myelin sheaths can result in devastating neuronal diseases, such as multiple sclerosis [3]. Even though myelin is formed mostly during postnatal development in mammals, the specification of oligodendrocytes cell fate occurs early in development [4]. Numerous studies have shown that this specification of oligodendrocyte progenitors depends on a single gene, *Olig2*(*NM_016967*) [5], [6]. *Olig2* is a basic-helix-loop-helix transcription factor and acts as a transcriptional repressor [7], [8]. It is structurally conserved from humans to zebrafish [6], [9]. Mice homozygous for *Olig2* deletion do not form oligodendrocytes, and die on the day of birth [6], [10]. *Olig2* misexpression has been associated with neuronal disorders, including schizophrenia [11], [12] and Alzheimer's disease [13]. Thus manipulating *Olig2* expression could have therapeutic potential for some neurodegenerative diseases.

The expression of *Olig2* is first detected in mice at E8.5 in the ventral portion of the mouse neural tube [6], [7]. This precedes the expression of early oligodendrocyte precursor markers such as *platelet-derived growth factor receptor α*, whose expression is first detected at E12.5 [7]. *Olig2* expression is maintained in mature oligodendrocytes, but not in astrocytes, in the adult central nervous sytem [14]. The basal promoter of *Olig2* has been located and is functional in cell types that do not express *Olig2* [15]. A motor neuron-specific enhancer has also been located downstream of *Olig2* based on transgenic mouse studies [16]. However, other factors involved in the molecular events that regulate *Olig2* expression remain to be elucidated.

To further our understanding of the mechanisms by which *Olig2* expression is regulated, we identified potential regulatory regions upstream of the *Olig2* coding region using criteria such as clustering of potential transcription factor binding sites and sequence conservation. We then verified one of these predictions by testing its effect on the expression of an *Olig2* reporter gene in mouse embryonic stem cells. Embryonic stem (ES) cells are an attractive tool for biomedical research since they have the potential to produce many different cell types, including neuronal precursors, *in vitro* [17]–[19]. Furthermore, the earliest stages of oligodendrocyte development in ES derived neuroepithelial cells follow a similar ordered sequence to that observed *in vivo* [15], [20]–[22]. ES cell derived oligodendrocytes also interact with host neurons and myelinate axons in the brain [23], [24]. By studying the effect of potential regulatory elements in both undifferentiated and differentiated states, we will be able to gain a better understanding of the regulation of *Olig2*. We suggest that bioinformatic identification of potential cis-regulatory elements coupled with rapid experimental verification in ES cells will provide a powerful combination for elucidating the transcriptional control of major developmental regulators.

## Results

### Potential regulatory regions identified computationally

The 150 kb sequence upstream of *Olig2* region has been shown to be sufficient to drive normal expression of *Olig2* [20]. We sought to identify the regulatory elements in this region, reasoning that potential regulatory elements were likely to share some of the following properties: high sequence conservation relative to orthologous sequences from other vertebrate genomes, stretches of sequence that are perfectly conserved between orthologous regions from mammalian genomes, and, high densities of potential transcription factor binding sites (TFBS) [25]–[30]. We therefore devised metrics to score these properties in the upstream region of *Olig2*.

**Figure 1:**
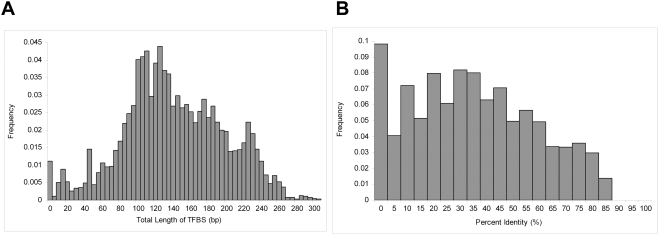
A) Frequency distribution of potential transcription factor binding sites in 2 Kb windows across the *Olig2* upstream region. Sequences upstream of *Olig2* were scanned using *Patser* [31], [32] for any potential binding site for any of the mammalian transcription factors in TRANSFAC [33]. Total lengths of potential TFBS were summed up for each 2 kb window. B) Frequency distribution of percent identity in the *Olig2* promoter. The average percent identity was calculated for every 2 kb window spanning the multiple alignments of sixteen species in the regions upstream of *Olig2.*

In order to identify regions upstream of *Olig2* that are dense in potential TFBS, we removed 57 kb of repeated sequence, leaving 93 kb of sequence in the analysis. We used the program *Patser* [31], [32] to identify sequences that match the Position Weight Matrix (PWM) models of all known mammalian TFBS from TRANSFAC [33]. The TFBS density in 2 kb regions was defined as the total number of base pairs contained in TFBS. The top 6% of windows contain at least 230 bp of TFBS **(** ***Figure 1A*** and ***Figure S1*** **)**.

We located evolutionarily conserved regions by calculating the percent identity in all 2 kb regions spanning the *Olig2* promoter using multiple alignments of seventeen species: mouse, rat, rabbit, human, chimp, macaque, dog, cow, armadillo, elephant, tenrec, opossum, chicken, frog, zebrafish, tetraodon and fugu. The average percent identity per 2 kb region is 35%. The top 8% of windows have an average percent identity of at least 75%. **(** ***Figure 1B*** and ***Figure S1*** **)**.

**Figure 2:**
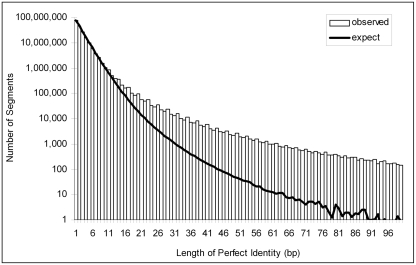
Expected and observed length distribution of non-coding sequences with perfect conservation across human, mouse, and rat reference genomes of *Olig2*. The expected length distribution was generated by randomizing the columns in the human, mouse, and rat multiple alignments containing only non-coding regions.

We also searched for ultraconserved elements in regions upstream of *Olig2*. Ultraconserved elements were first defined as sequences at least 200 bp long that show perfect conservation in alignments of the human, mouse and rat reference genomes (perfect HMR conservation). They have been hypothesized to represent sequences under selection for specific functions [34], [35] . The 200 bp threshold used to define these elements is arbitrary and we therefore sought to determine empirically what the appropriate threshold length should be for defining a sequence as ultraconserved. To build an expected length distribution of segments with perfect conservation, we assumed that the identity of each base was independent of all other bases and randomly swapped the columns in alignments of non-coding portions of the three genomes. For each length, we tabulated the number of perfectly conserved sequences from the simulation and compared it to the observed number of segments in the actual alignments **(** ***Figure 2*** **)**. A sequence with 38 bp of perfect HMR conservation was statistically significant (280 segments expected, 8031 observed, false positive rate<0.05). Using this threshold, we found sixteen such sequences upstream of *Olig2* (range 38–106 bp) **(** ***Table 1*** and ***Figure S1*** **)**.

**Table 1:** Perfectly conserved sequences upstream of *Olig2*.

Start Coordinate (Feb 2006)	Stop Coordinate (Feb 2006)	Length (bp)
90,986,285	90,986,328	44
90,994,033	90,994,087	55
90,994,103	90,994,146	44
90,994,165	90,994,225	61
90,994,290	90,994,338	49
90,994,531	90,994,576	46
91,014,404	91,014,444	41
91,029,418	91,029,523	106
91,029,525	91,029,569	45
91,062,100	91,062,137	38
91,062,232	91,062,269	38
91,062,462	91,062,506	45
91,081,445	91,081,489	45
91,097,962	91,098,002	41
91,098,179	91,098,216	38
91,112,651	91,112,711	61

Sequences showing perfect identity over at least 38 bp across human, mouse, and rat reference genomes are identified in the *Olig2* promoter. This length threshold of 38 bp was selected based on a comparison between expected and observed length distributions of perfectly non-coding conserved segments across the entire genome alignments.

The analyses described above identified 2 kb sequence windows upstream of *Olig2* that either contained a high density of potential TFBS, showed high conservation across many vertebrate genomes, or showed stretches of perfect HMR conservation. We combined these sets of sequences to identify potential regulatory regions **(** ***Table 2*** **,** ***Table S2*** **)**. Some regions that did not included stretches of identical bases, but were otherwise highly conserved, were also included.

**Table 2:** Putative regulatory regions in the *Olig2* promoter.

Start Coordinate (Feb. 2006, mm8)	Stop Coordinate (Feb. 2006, mm8)	Average percent identity	Total length of Potential transcriptional factor binding sites (both strands, bp)	Total length of segments with perfect conservation (bp) [number of segments]
90,982,736	90,984,735	0.708	264	0
90,987,057	90,989,057	0.778	191	0
90,992,944	90,994,943	0.826	236	255 [5]
91,002,382	91,004,381	0.780	243	0
91,028,363	91,030,362	0.803	225	151 [2]
91,051,171	91,053,170	0.762	260	0
91,061,447	91,063,446	0.752	221	121 [3]
91,080,626	91,082,625	0.837	230	45 [1]
91,097,264	91,099,263	0.710	301	79 [2]
91,112,409	91,114,408	0.747	237	61 [1]

Top candidates contain stretches of perfectly conserved sequences from human, mouse, and rat alignments, are highly conserved across at least four species, and contain large number of TFBS.

The *Olig2* basal promoter has been shown to reside in the 2 kb region immediately upstream of the *Olig2* exons [15], [20]. This region also appeared on our candidate list (*mm8_chr16: 91*,*112*,*409 – 91*, *114*,*408*; ***Table 2***), suggesting that our criterion represented characteristics of known regulatory regions. This 2 kb region contains 237 bases covered by potential TFBS, many of which were binding sites for *Sp1*. *Sp1* is a transcription factor that activates gene expression [36], [37]. In addition, this region has 74.7% conservation across multiple species and includes a 61 bp sequence of perfect HMR conservation.

Another region on our candidate list (*mm8_chr16: 91*,*080*,*626 – 91*,*082*,*625;* ***Table 2***) may contain a repressive element (DIG, unpublished data). This region shows 83.7% conservation across multiple genomes, the highest in the *Olig2* promoter, and includes a 45 bp sequence of perfect HMR conservation. A recent study demonstrated that this region has different methylation patterns in ES cells and neuronal precursor cells [38]. Furthermore, this region contains numerous binding sites for Gut-Kruppel-Like Factor 4 (*Gklf4*). Recent studies have shown that *Gklf4* is one of four transcription factors required to induce pluripotent stem cells from mouse fibroblasts [39]. There is also evidence that *Gklf* plays important roles in ES cells, such as in inhibiting myofibroblast differentiation [40] and regulating the expression of *Lefty1* [41]. We therefore suspect that *Gklf4* plays a similar role in regulating oligodendrocyte differentiation by repressing *Olig2* expression in undifferentiated cells.

In addition, another region on our candidate list (*mm8_chr16:91*,*061*,*447 – 91*,*063*,*446*, ***Table 2***) was shown to have different methylation patterns between ES cells and neuronal precursor cells [38]. This region contains three segments of perfect HMR conservation with a combined length of 121 bp. It has 75.2% identity across all genomes in the alignment and contains 221 bp of potential TFBS. We also speculate that this region contains sequences that regulate *Olig2* expression.

Our top candidate for a new regulatory region (*mm8_chr16: 91*,*028*,*363-91*,*030*,*362;* ***Table 2***) contained a sequence we designated as *ULTRA* (*chr16 91*,*029*,*26-91*, *029*,*835*, ***Table S2***). It contains 106 bp and 45 bp sequences of perfectly HMR conservation, which are separated by only a 1 base mismatch in the human genome. This region also has 80.3% conservation among the set of seventeen vertebrate genomes and was significantly enriched for potential TFBS. In addition, *ULTRA* shows 71% sequence similarity with another region (*mm8_chr10:18*,*999*,*620-18*,*999*,*731*) that also shows perfect identity among human, mouse, and rat genomes. This second region is located 46 kb upstream of *Olig3*, a paralogous gene of *Olig2*. The presences of these similar highly conserved elements in the promoters of two genes in the *Olig* family suggest that these elements play a role in *Olig* gene expression. We, therefore, tested whether *ULTRA* could influence the expression regulated by the *Olig2* promoter in the current study.

### *ULTRA* represses *Olig2* basal promoter regulated expression in embryonic stem cells

We constructed three plasmids (*P-plasmid*, *UP-plasmid*, *PGK-plasmid*) to test the regulatory activity of *ULTRA* (Methods). All three constructs all included a neomycin cassette, which confers resistance to G418, and the coding region of green fluorescent protein (GFP), but differed in the sequences upstream of GFP. The *Olig2* basal promoter sequence was placed upstream of GFP in *P-plasmid*. The *UP-plasmid* is identical to the *P-plasmid* except that the *ULTRA* sequence is included directly upstream of the *Olig2* basal promoter, and is designed to test whether the *ULTRA* region can modulate the activity of the *Olig2* basal promoter. The phosphoglycerate kinase promoter was placed upstream of GFP in *PGK-plasmid* to serve as a positive control.

**Figure 3:**
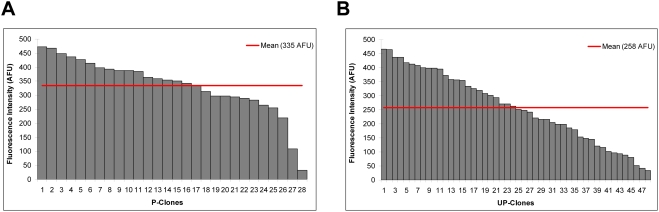
A) Distribution of fluorescence intensity in 28 *P-clones* in the undifferentiated state. B) Distribution of fluorescence intensity in 48 *UP-clones* in the undifferentiated state. *P-* and *UP-clones* were created by transfecting ES cells using two different constructs: one with a basal *Olig2* promoter upstream of GFP and the other with *ULTRA* placed in front of the *Olig2* promoter and GFP. Integration events were selected by applying neomycin. Clones were expanded and analyzed for GFP expression by flow cytometry. The distributions of fluorescence intensity for *P-* and *UP-clones* were statistically different (P-value<0.05, Wilcoxon rank sum test).

The three constructs were separately transfected into mouse ES cells and random integration events were selected for using G418. The resulting cell lines were designated *P-clones*, *UP-clones*, and *PGK-clones*. Polymerase chain reaction (PCR) was used to verify that the complete sequences including the promoter and the GFP were integrated into the genome (data not shown). We kept only clones with intact target sequences, resulting in 28 *P-clones* 48 *UP-clones* and 26 *PGK-clones*. These clones were grown on STO feeder cells and then assayed by flow cytometry to measure GFP expression. To distinguish ES cells from these STO cells in each culture, we incubated all cells with a monoclonal antibody to mouse SSEA-1 (Stage-specific embryonic antigen-1), which is expressed on the surface of ES cells, but not on STO cells. The binding events of SSEA-1 antibody were detected using an anti-mouse secondary antibody conjugated to Alexa Fluor 555. The fluorescent levels of GFP and the Alexa Fluor 555 antibodies for each clone, along with the sizes of individual cells, were simultaneously detected on a flow cytometer. We included four wells of STO cells and four wells of ES cells as controls. These controls were used to build a statistical model to distinguish ES cells from non-ES cells in *PGK-clones*. We built a logistic regression model to predict cell type (ES vs non-ES) with three variables: antibody fluorescence, cell granularity measured by side scatter values, and the plate in which the clone was located. With this model, we first assessed the GFP expression of *PGK-clones*. The GFP expressions of *PGK-clones* range from 87.76 to 656.90 arbitrary fluorescent units (AFU) after adjusting for background fluorescence, indicating that the GFP sequence was functional and that integration sites in the genome indeed influence expression. We chose one *PGK-clone* with the smallest standard deviation in GFP expression to include on plates containing the *P-* and *UP-clones*. This *PGK-clone* serves as a technical positive control for each plate. We also used a similar logistic regression to differentiate between ES and non-ES cells in *P-* and *UP-clones*. With this model, a threshold of 0.48 was used to differentiate between ES and non-ES cells. This threshold correctly predicted 92% ES cells and 97% of STO cells in the control wells. This model was applied to all *P-* and *UP-clones* and only cells deemed to be ES cells were kept for further analysis. After removing all the non-ES cells from each clone, the average number of cells per *P-clone* is 151,217 cells, ranging from 58,770 to 196,069 cells. The number of cells per *UP-clone* ranged from 47,182 to 193,392 cells with an average of 134,618 cells per clone. The mean GFP expression for *P-clones* was 335 AFU, whereas the mean GFP value for *UP-clones* was 258 AFU, after correcting for background fluorescence **(** ***Figure 3*** **)**. The reduction in GFP expression in the *UP-clones* relative to the *P-clones* was statistically significant (*P* value<0.05, Wilcoxon rank sum test), suggesting that *ULTRA* represses the expression driven by the *Olig2* basal promoter.

### *ULTRA* does not affect *Olig2* basal promoter regulated expression in differentiated neural precursor cells

**Figure 4:**
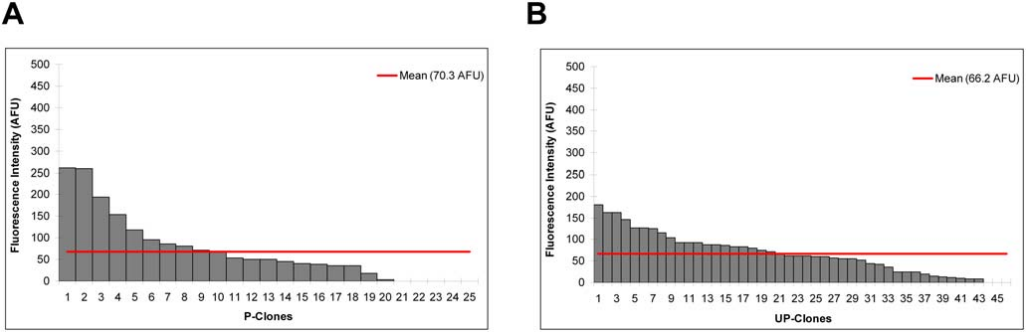
A) Distribution of fluorescence intensity in 25 *P-clones* in the differentiated state. B) Distribution of fluorescence intensity in 46 *UP-clones* in the differentiated state. ES cells were grown in suspension to form embryonic bodies and retinoic acid and Shh agonist Hh-Ag 1.4 were added to the medium after 2 days. Differentiated cells were detected using *Olig2* antibodies. The distributions of differentiated *P-* and *UP-clones* were not statistically different (*P* value >0.05, Wilcoxon rank sum test).

We investigated the possibility that this *ULTRA* also affects expression driven by the *Olig2* basal promoter in differentiated cells. We differentiated all 76 ES clones into neural precursor cells by treating them with retinoic acid and a Sonic Hedgehog agonist. 25 *P-clones* and 46 *UP-clones* survived differentiation. Differentiation induces *Olig2* expression and differentiated cells were selected based on labeling with *Olig2* antibodies. We assayed GFP fluorescence and *Olig2* staining for each clone using a flow cytometer. We defined differentiated cells from each clone as those with higher *Olig2* antibody fluorescence values than the undifferentiated ES cells. We computed their average GFP expression of each differentiated clone by subtracting the background fluorescence determined from the control wells on the same plate. The mean GFP expression for *P-clones* was 70.3 AFU and for *UP-clones* was 66.2 AFU **(** ***Figure 4*** **)**. The difference in GFP was not statistically significant (*P* value >0.05, Wilcoxon rank sum test), indicating that *ULTRA* does not affect the expression driven by the *Olig2* basal promoter in neuronal precursors.

## Discussion

Despite mounting evidence that the regulation of *Olig2* at the level of transcription plays an important role in glial cell fate specification, we know little about the regulation of *Olig2*. Here we presented a preliminary study aimed at identifying cis-regulatory regions that control *Olig2* expression. Since distal cis-regulatory elements in vertebrates can be located far from the gene [42], we scanned the 150 kb of *Olig2* upstream sequence and identified ten regions containing potential regulatory elements which might play a role in controlling *Olig2* gene expression. Here we verified one candidate region in mouse ES cells and neuronal precursor cells. Our results indicate that this region contains sequences that repress *Olig2* expression in undifferentiated cells. In neuronal precursor cells, this region does not appear to repress *Olig2* expression. This element may play an important role in keeping *Olig2* expression off in undifferentiated ES cells. Future studies, such as deleting this region from its native locus, are needed to further elucidate the molecular roles of this region.

Our results suggest a model by which *Olig2*, a key developmental transcription factor, is expressed only in cells developing along the neural lineage. The basal promoter of *Olig2* shows constitutive activity in both undifferentiated ES cells and neuronal precursors [15], [20], indicating that the basal promoter alone cannot explain *Olig2* expression. The expression of *Olig2* is likely repressed in undifferentiated cells through the concerted action of upstream repressive elements including the *ULTRA* and, possibly, two other regions [38]. This repression is then relieved as cells develop along the neuronal lineage allowing *Olig2* expression. The predictions of this model are testable and provide a framework for further studies of *Olig2* expression regulation.

The recent discovery of ultraconserved elements [34] has generated a debate on the extent to which sequence conservation reflects functional importance. Ultraconserved elements are more conserved than coding sequences, thus prompting the suggestions that they encode essential functions. Here we used an empirically defined threshold to identify elements with perfect identity that we would not expect to arise randomly throughout evolution. The *ULTRA* region we tested in this study contains two such elements, but the region only has a mild repressive effect on expression. These results are in agreement with our previous study, which demonstrated that selection on these elements with contiguous identity is only weakly purifying [35].

We identified ten potential regulatory regions of *Olig2* using a combination of sequence conservation across seventeen vertebrate genomes and TFBS density, and perfect HMR conservation. Only one of these candidate regions, the *Olig2* basal promoter, had previous, direct experimental support to suggest that it regulates *Olig2* expression [15], [20]. Two candidate regions have methylation profiles that suggest a regulatory role. Here we present direct evidence that a fourth candidate region, *ULTRA*, modifies the expression regulated by the *Olig2* basal promoter in a manner consistent with our current understanding of *Olig2* expression during oligodendrocyte differentiation. This study provides a basis for studying *Olig2* regulation on the molecular level. We anticipate that further studies on our predicted regulatory regions will continue to unravel the regulatory interactions governing the timing and location of *Olig2* expression during development.

## Materials and Methods

### Computational Predictions of Regulatory Elements

We predicted regulatory elements using three criteria: density of potential transcription factor binding sites (TFBS), sequence conservation across multiple genomes, and stretches of sequences with perfect conservation in human, mouse, and rat reference genomes.

The 150 kb sequence upstream of the mouse *Olig2* gene was downloaded from the UCSC Genome Browser with nucleotides in repeat regions masked (*mm8_90*,*964*,*409-91*,*114*,*408*) [47]. We used *Patser* [31], [32] to scan this region for any potential binding site for any of the 263 mammalian transcription factors in TRANSFAC [33]. The numbers of potential sites overlapping each nucleotide position were summed up for individual bases and then the density of potential binding sites for each 2 kb window along the 150 kb upstream of the *Olig2* coding sequences by adding up the number of sites at each base in the window.

To identify highly conserved regions, multiple sequence alignments with seventeen species spanning 150 kb upstream regions of *Olig2* were downloaded from the UCSC Genome Browser [47]. The seventeen species include mouse, rat, rabbit, human, chimp, macaque, dog, cow, armadillo, elephant, tenrec, opossum, chicken, frog, zebrafish, tetraodon and fugu. To narrow down the conserved regions in this set of alignments, the percent identity for each base found in the mouse genome was calculated using the following scoring scheme: a percent identity of zero was assigned to a base if fewer than three species, other than mouse, contained a nucleotide in that position; otherwise, the percent identity is calculated by dividing the total number of species having the major allele by the total number of species having a non-gap base in that position. We then calculate the average percent identity for overlapping windows of 2 kb along the *Olig2* promoter.

The third criterion we used to predict the regulatory elements is the presence of sequences with uninterrupted perfect identity across human, mouse and rat, so-called ultraconserved elements. We empirically determined the length threshold for a significant run of perfectly conserved adjacent residues across human, mouse and rat genomes. We used the same human-mouse-rat alignments that Bejerano et al. [34] used in first identifying the ultraconserved elements to generate the empirical length distribution. We removed all exonic sequences from the alignments and then randomized non-gapped columns in each alignment. After each round of randomization, the frequencies of lengths of perfectly matched sequences were recorded. Ten complete randomizations were done across all alignable regions of the three genomes and the resulting frequencies were averaged to create the empirical length distribution of perfectly conserved sequences. We selected the threshold length, 38 bp, by using a false positive rate of 0.05 derived from comparing the expected distribution with the observed. A false positive rate was calculated as the number of expected segments divided by the number of observed segments of the same length. We then scanned the 150 kb upstream sequence of *Olig2* for any identical sequence across human, mouse, and rat genomes whose length was equal or greater than the threshold length.

The top candidate regions with potential regulatory functions were selected such that they contained perfectly matched sequences, were highly conserved among all species in the alignment and had dense transcription factor binding sites. Regions were also considered as candidates when they included high numbers of binding sites and were highly conserved only. In total, ten regions of 2 kb each were identified as potential regulatory regions.

### Construction of Enhancer Plasmids

We chose one computational prediction to validate experimentally in mouse embryonic stem (ES) cells: *mm8_chr16: 91*,*028*,*363 — 91*,*030*,*36*. To test whether this region contained any element that has enhancer activity, the most conserved sequence within this region, *mm8_chr16: 91*,*029*,*261 — 91*, *029*,*835* (designated *ULTRA*), was chosen to test for its activity in ES cells. We constructed three plasmids from the *pEGFP* plasmid (Clontech): a plasmid with a native promoter from the *Olig2* locus in front of the green fluorescence protein (GFP) gene (*P-plasmid*), a plasmid with *ULTRA* cloned in front of the native promoter, followed by the GFP gene (*UP-plasmid*), and a plasmid with the phosphoglycerate kinase as promoter in front of the GFP gene (*PGK-plasmid*).

The *P-plasmid* was constructed by cloning the 2 kb sequence in front of the first exon of *Olig2* (*mm8_chr16:91*,*112*,*391 — 91*,*114*,*400*) upstream of the GFP gene on the *pEGFP* plasmid. This sequence was shown to contain the Olig2 basal promoter [15], [20]. The sequence was amplified from the mouse genome using primers listed in ***Table S1***. The polymerase chain reaction (PCR) products and the *pEGFP* plasmid were both digested with *BamHI* and *Sal1* restriction enzymes. Ligation was carried out at 16°C for one hour. The *UP-plasmid* was constructed by inserting the *ULTRA* upstream of the 2 kb segment. The *ULTRA* was amplified from the mouse genome using appropriate primers **(** ***Table S1*** **).** The PCR product and the *P-plasmid* were then digested with *BglII* and *HindIII* restriction enzyme and ligated at 16°C for one hour. The *PGK-*plasmid was constructed by putting the phosphoglycerate kinase as a promoter upstream of GFP. The phosphoglycerate kinsase sequence was digested from *pBC293* (Cohen Lab, Washington University) using *EcoRI* and *BglII* restriction enzymes. The *pEGFP* vector was then digested with these two enzymes and the ligation reaction was carried out at 16°C in one hour. Transformation of *E. coli* was selected with 30 mg/mL kanamycin.

### Embryonic stem cell culture and transfection

The ES cells used in this study were the mouse RW4 line and were cultured in conditions previously described [20]. About 5 ug of *P-plasmid*, *UP-plasmid*, and *PGK-plasmid* were used in independent electroporations to created *P-clones*, *UP-clones* and *PGK-clones*. Electroporations were carried out using program A-013 on a Nucleofector II device (Amaxa Inc.) with the provided ES cell electroporation solution. Electroporated cells were transferred to a 100 mm gelatinized dish with 10 mL of complete medium (CM) (DMEM+10% fetal bovine serum, 10% newborn calf serum and nucleoside supplement) with 1000 U/mL leukemia inhibitory factor (LIF), 0.1 mM β-mercaptoethanol [22]. 50 uL of 250 ug/mL of G418 (GIBCO) was added to the tranfected cells to select for cells with a stably integrated transgene. After eight days, clones resistant to G418 were transferred into 96-well plate wells with about 30,000 STO cells as feeder layers. STO cells had been irradiated with 3500 rads to prevent replication. ES cell clones were then cultured and expanded in CM as described until each clone could be maintained and harvested from a well on a six-well plate [20].

### PCR verification of integration events

The cells were washed with Dulbecco's solution A (PBS), dissociated with 380 uL 0.25% Trypsin-EDTA (GIBCO), and harvested from six-well plates. DNA was extracted from cells using a DNA purification kit (5 PRIME ArchivePure, Fisher Scientific). To verify the complete sequence from the beginning of the promoter to the end of the GFP gene on the *P-plasmid* was integrated into the genome, two separate PCR reactions were performed using two primer pairs **(** ***Table S1*** **)**. Similarly, *UP-clones* and *PGK-clones* were verified via PCR reactions **(** ***Table S1*** **)**.

### Plate layout and antibody staining

Each clone was placed into a well on a 96-well plate and GFP expression in clones was assessed by flow cytometry. To control for plate-to-plate variation, four sets of three wells each (A1–A3, A10–A12, H1–H3, H10–H12) were used as control wells on each 96-well plate. Each set of three control wells contained either STO cells, RW4 cells, or *PGK-clone* cells, in that order. The *PGK-clone* was used as a positive control. Each clone with approximately four to five million cells were washed with PBS, dissociated with 380 uL 0.25% Trypsin-EDTA, and recovered with 1.6 mL CM. Each clone was then spun down at 1200rpm for 5 min, resuspended in 100 uL PBS, and transferred into a well on a 96-well plate. Each clone was washed with 100 uL PBS twice. The cells were then incubated at 4°C with 50 uL of unconjugated mouse SSEA-1(Stage-specific embryonic antigen-1) antibodies for 20 minutes (Millipore, 1:50 dilution). SSEA-1 is present only on the cell surface of ES cells, but not STO cells. After incubation, the cells were washed twice with 100 uL PBS. We then stained with 50 uL of anti-mouse IgM antibodies (Invitrogen, 1:100 dilution) for 20 minutes at 4°C. These secondary antibodies were conjugated to Alexa Fluor 555. The cells were washed twice with 100 uL PBS and then resuspended in 150 uL of PBS for fluorescence analysis on a flow cytometer.

### Analysis of GFP Expression in undifferentiated cells by flow cytometry

Linear and log values of fluorescence level at 510 nm (GFP fluorescence) and 565 nm (fluorescence of Alexa Fluor 555 antibodies), as well as forward scatter (*FS*) and side scatter (*SS*) values were obtained for each cell using a Beckmann-Coulter Cytomics FC500 MPL with a 488-nm laser. The Alexa Fluor 555 antibodies bind to SSEA-1 primary antibodies, which recognize the antigens that are present only on the surface of ES cells. To select only the undifferentiated ES cells from each well, the fluorescence values of antibodies gathered from the RW4 cells and STO cells in all the control wells were used to construct a logistic regression model, which gives the probability of a cell being an ES cell. With the assumption that majority of the RW4 cells were undifferentiated and STO cells were not ES cells, we expected the RW4 cells and STO cells to show different distributions of antibody fluorescence values. We chose the best model, based on the lowest Akaike information criterion (AIC) [48], to predict the membership of each cell (a ES cell or non-ES cell). Parameters tested in the model included: *Plate* effect, *log*(*antibody fluorescence*), *log*(*FS*), *log*(*SS*), *antibody fluorescence*, *FS*, and *SS* values. We conducted leave-one-out cross validations to assess the sensitivity and specificity of the model. Sensitivity was defined as the number of correctly predicted ES cells divided by the total number of true ES cells. Specificity was defined as the number of correctly predicted non-ES cells divided by the total number of true non-ES cells. We chose a threshold value for selecting ES cells based on the sum of sensitivity and specificity values. To investigate whether the location of each clone on a plate affects the fluorescence levels of cells, models built using only three sets of the STO-RW4 control wells were used to predict the cell types of cells in the set of control wells that were not included in the models. The sensitivity and specificity of the predictions were then compared among the four different sets of wells. The appropriate logistic regression model was used to select the undifferentiated cells from each clone on the plates. To test whether there is a difference in the distribution of mean GFP fluorescence levels among *P-*and *UP-clones*, the Wilcoxon rank sum test was used.

### Differentiation of ES cells

ES cells were treated with a protocol that induces ventral neural cell fates. ES cells are first scrapped off the bottom of flasks and cultured for two days in M-DFK5 medium [20]. After two days, embryonic bodies were plated in a 24-well plate in the same media in the presence of 2 µM retinoic acid (Sigma) and 30 nM of Shh agonist Hh-Ag 1.4 (Curis) for 4 days.

### Plate layout and antibody staining

We reserved four sets of three wells on each plate to serve as controls. Each set of control wells contained a well of undifferentiated RW4 cells, differentiated RW4 cells, and differentiated TG25 ES cells. TG25 cells carry a GFP knock-in at the *Olig2* genes; GFP in the cells is turned on when they were differentiated [49], thus serving as a positive control. The cells were moved from 24-well plates to 96-well plates prior to staining by using the procedures described earlier. After washing with PBS twice, we fixed the cells with 4% paraformaldehyde for 30 minutes at room temperature. We then permeated the cells with 0.1% triton-X for 10 minutes and blocked the cells with bovine serum albumin for 30 minutes. Cells were washed with PBS between each step. We stained the cells with *Olig2*-antibodies (Chemicon, 1:100 dilution) for 30 minutes at 4°C. The second staining was done using the Alexa Fluor 555 goat anti-rabbit IgG (H+L) antibody (Invitrogen, 1:100 dilution) and incubated for 30 minutes at 4°C. These conjugated secondary antibodies bind to *Olig2*-antiboies. After staining, the cells were then resuspended in 150 uL of PBS for fluorescence analysis on a flow cytometer.

### Analysis of GFP Expressions in differentiated cells by flow cytometry

To select only the differentiated cells from each well, the fluorescence values of Alexa Fluor 555 antibodies emitted from the undifferentiated and differentiated RW4 cells on each place were used. The mean and standard deviation of the antibody fluorescence from the undifferentiated RW4 cells were calculated. The 95% confidence intervals were used as a threshold to distinguish differentiated cells from undifferentiated cells. Any cell with a fluorescence value higher than the 95% upper bound was deemed differentiated. This threshold was applied to all clones on a plate and GFP fluorescence was calculated for the differentiated cells only. The average GFP values for the differentiated RW4 cells were subtracted from the GFP values for each clone on the same plate to control for plate-to-plate variation. We used the Wilcoxon rank sum test to test for differences in the distribution of mean GFP fluorescence levels among *P-*and *UP-clones*.

## Supporting Information

Figure S1 Potential transcriptional factor binding sites (TFBS) density, conservation level and perfectly conserved human-mouse-rat sequences in Olig2 promoter. The locations of potential TFBS were identified using the program Patser [31], [32] and all known mammalian transcriptional factor matrices in TRANSFAC [33]. The level of conservation was calculated using multiple alignments of seventeen vertebrate genomes. Sequences with at least 38 bp of contiguous perfect conservation across the human, mouse, and rat genomes were also located in the Olig2 promoter. Bases are colored according to the level of conservation and the number of potential TFBS located. Bases with high sequence conservation or contain high number of potential TFBS are represented by darker blue whereas bases with low sequence conservation or contain low number of potential TFBS are represented by lighter blue. Candidate regions with regulatory potentials are indicated with red boxes.
(1.55 MB TIF)

Table S1 List of Primers
(0.03 MB DOC)

Table S2 Sequence of the candidate region.
(0.09 MB DOC)
