# Supplementary figures and images for: Ultraconserved Elements in the Olig2 Promoter
Source: PLoS One. 2008 Dec 16;3(12):e3946. doi: 10.1371/journal.pone.0003946 (PMC2596485; doi:10.1371/journal.pone.0003946)

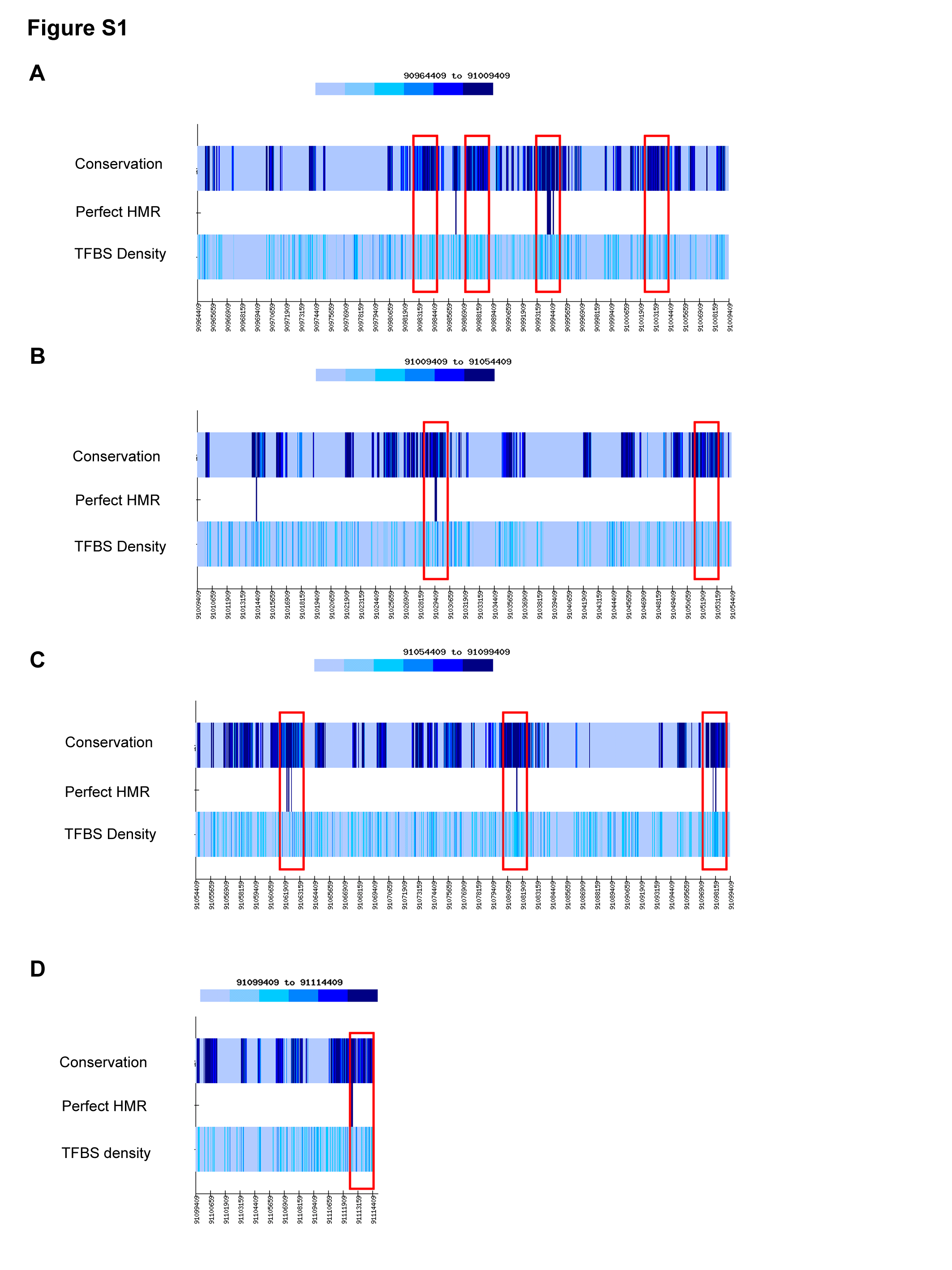

Supplement: Figure S1 — Potential transcriptional factor binding sites (TFBS) density, conservation level and perfectly conserved human-mouse-rat sequences in Olig2 promoter. The locations of potential TFBS were identified using the program Patser [31], [32] and all known mammalian transcriptional factor matrices in TRANSFAC [33]. The level of conservation was calculated using multiple alignments of seventeen vertebrate genomes. Sequences with at least 38 bp of contiguous perfect conservation across the human, mouse, and rat genomes were also located in the Olig2 promoter. Bases are colored according to the level of conservation and the number of potential TFBS located. Bases with high sequence conservation or contain high number of potential TFBS are represented by darker blue whereas bases with low sequence conservation or contain low number of potential TFBS are represented by lighter blue. Candidate regions with regulatory potentials are indicated with red boxes. (1.55 MB TIF) [file pone.0003946.s001.tif]
