# Supplementary material for: Ultraconserved Elements in the Olig2 Promoter
Source: PLoS One. 2008 Dec 16;3(12):e3946. doi: 10.1371/journal.pone.0003946 (PMC2596485; doi:10.1371/journal.pone.0003946)
Supplement: Table S1 — List of Primers (0.03 MB DOC) [file pone.0003946.s002.doc]

**Table S1 List of Primers**

| Region | Primer Sequence (5’—3’) |
| --- | --- |
| *mm8_chr16: 91,112,391 — 91,114,400*  (Olig2 Basal promoter) | Forward: GCATGTCGACGGGTGGCTGCAACCTATCTCCCGCATATTGTACCGCCTGAGG  Reverse:  GCATGGATCCGCTGGGTGGAGGCAGCAGCGACGGC |
| *mm8_chr16: 91,029,261 —*  *91,029,835*  (ULTRA) | Forward:  CGCGCGAGATCTCATAAACACATAGATACCGAAGAGTTAATTTGCGTTTTTTAAACCCCC  Reverse:  CGCGCGAAGCTTTCTTGCTCCATTCAGCCTCCTGAGAATGTTGGGTCG |
| *P-clones* verification | 1) Forward:  GCATGTCGACGGGTGGCTGCAACCTATCTCCCGCATATTGTACCGCCTGAGG  Reverse:  GCCGTAGGTGGCATCGCCCTCGCCC  2) Forward:  GCATGGATCCGATTCCCCGTCTCACTCCGTACCCTGGCC  Reverse:  GCTTTACTTGTACAGCTCGTCCATGCCGAGAGTGATCC |
| *UP-clones* verification | 1) Forward:  CTAGACTTCCCCAGGCAATTCACCCAGATCC  Reverse:  GCTTTACTTGTACAGCTCGTCCATGCCGAGAGTGATCC  2) Forward:  CGCGCGAGATCTCATAAACACATAGATACCGAAGAGTTAATTTGCGTTTTTTAAACCCCC  Reverse:  GGCCAGGGTACGGAGTGAGACGGGGAATC |
| *PGK-clones* verification | Forward:  CCTACCGGGTAGGGGAGGCGCTTTTCCCAAGG  Reverse:  GCTTTACTTGTACAGCTCGTCCATGCCGAGAGTGATCC |
