# Supplementary material for: Ultraconserved Elements in the Olig2 Promoter
Source: PLoS One. 2008 Dec 16;3(12):e3946. doi: 10.1371/journal.pone.0003946 (PMC2596485; doi:10.1371/journal.pone.0003946)
Supplement: Table S2 — Sequence of the candidate region. (0.09 MB DOC) [file pone.0003946.s003.doc]

**Table S2 Sequence of the candidate region.**

| Start  Coordinate  (Feb. 2006, mm8) | Stop  Coordinate  (Feb. 2006, mm8) | Sequence |
| --- | --- | --- |
| 90,982,736 | 90,984,735 | AACCACAGCAAACAAATCCCATCTTTGCTGTAATTATTACAAACTATAAG  GCAGTGTGGGGTGAGGACTAGAGGGGGGAAGTAGCGTGAAGGCCACTGAA  CAGCCTTCTTTTACTTTGGTGACATTGGGCCCAGAATTCCACACTGCTGG  AAGCCACCTAATGCCCCTTAGCTTTTAGAATTATTGTGTCTTCACAGTAT  GCATTTGTAAGTAGAAGAGTAAAATAAAACACTAGCAAACAGGTCAGAAG  TTTGTTTAAAAAATACCATAGGAGTGCAAGAATTGTTTAATACCATCACT  CACCATCTTAGAAAAGCCCGTTATTATATATTTGGAAGCCCAGATGGTTG  TTTGTAAAACTCTAAAAACACTTTGTTTTACTTTAAAAGTTTTAATTAGG  AACAGACACTACACATTCACATGGAAACACAATGACCAAAAATGTACTAC  AGTGTTTCATGTTGAAATGCTAAGGGCATCTACATATATAGTCAGATAAA  ACTGGCCACCATCGAGACTACTGGAGCTAGTTCTAGAAGTACTATTAATT  CAACTGGACAAGTGAAACCTACCACAGACTCCAGCAGATAATTATTTATA  GATAACATTATAAATCCCTGACATTTATAAAAACTTATTGGAAAACTGTT  AGGAACTAAGGTACCTAAATAAAATGCCTCCTGTAAGCAAAGCTATAAAA  AAAAAATAGGTTTCTTGCATATGAATACAAAACAAAGTCAATCATTTCTC  AGCCTTATTCAACAGATGATCTTCCCCCTGTCCCCACCCTGCCCCCACCC  CACCACAGGCTCGATCCCTTCTTGCCTCTGAGCAGTGCTTTCACAGGAAG  GATTCTCTTGGATCAGGCGCAGTGGCGGCGAGGAACAAGGCAATCCTTGG  ACACACACCATTTTGTAGTTTGGGTTACCCGCTGGCAGGAACCAAGCATG  TGTTGGCTCCGTTTGGGCCATGTGTGTCAAACAAGAGCCTTTGAAGTTGT  TCAAATCAAGAGGATTAGCCATAGTTCTAGAGAGAGGCAGAGTTTGTTCC  TATGGCTTCACGTACCTGTTAACATGACTTAGATGCGTTTCCTCGTGGCA  ACGTGGCTATTTGAGGCTCTGTTTGCCTGAAAACAGCCATAGATTTAACA  GTTTCTTTGTGGCACTAGACCTCTTGAAACCATAAAAAGAAGGTATTCGA  TTATTATCTTAATCGCTCATCTAACACAGAGGGCAGGACCATCTGGGCAC  CGAGTTACTTCTCGGATCACTAAGTTTATGCACAAACCCCATGCGGTAAG  CTGCCTTTCTGACCTGCCACGCCATCCAGCCTGTTGCTTTGTGATTGGCT  GTGTGTAATGGTTCACTTTTTACTTAAAATGGGCTGTAAGGAGTAAGGCT  GTATTTGATGACATTGAGAAAATAGTGTTCATGGCATAAGCTGGCCAAAC  AGAGAAACATTTACGAAAGCTTTTAATTTAACAAAATATTGATGCCTTTG  AGATGACTCTTAGATTTATTCTGAAAATCAGCATCGAGATTTCTGAGTGA  TTCGGTATTGATTTCACAACAAGTATTTCATGAAATTTAAGAAACAAAAA  CTATAAATACATTACTATTAGAAAACACATCGTTTTGTGCTTTTGGCAGG  CATATTAACCAAAGAGGGGAGAACTTTGATTTGACCATTACACGATCATT  ACATTATTAGCCTTCGGACATTTTTGAAAGATGATTTCGTGGTCACTGTT  GCTTCTCTAAGAATTTTGTTAAAGGAGCCACAAAAGCATAAAAGCATCCT  TTACATAGCACAGCTGAGGGCATTTAACTTGTCAAAACGCTCAATGTCTA  GATCTACTTAGGAGCCTGTCCAGTCATTTTTCTGGTTAAAAGGTTTGTGG  CAGATGCAACTCTTTGTCCCCCTTTGCTCCCTCCTCCTCCTCCTCAGTTG  GATTTGGATTGTTTTACATAAACCACAAAATAAAAATACATAAATTACTT |
| 90,987,057 | 90,989,057 | GTACACTTTATGAAGGATGAAATACGACGAAACCGTATGATGCTAGCAGA  GGCAAGCCAATGAGTCAAGGTAGAAGTCAGTGTCCCCCGGCAACTAGAGG  AACGGCAGTGTCGGTTGTCAGATTTTCTGGCTCGAGGAAGGGTCAGGGAA  ATTTTGGGTCATTGGGTCACACCATCCGGAAGATCCCCGTATCCACCAGG  AAGCCCATCAGGTACCATGTAAAATCAGTTCCCAGGGAGGCACCATGCCA  ATAACAGTTGTTACTACAGGGCAATTGGTAAGATATCAATTGCTGTGACT  TTCACCCTTTAAGAATCGATTTTTTAAAAATACATTAAAAAGCAGATCCC  GCCCTGCTTTCTAACAACAACGACTACAACAACAACTTTTGTTTCCGGGT  GCCCTTGGGCTGCAATGGTTCTATTCCCTTTTGGGTAGAGATAAGAAGGG  GACGATGAATCTGACGCTTCTTCCCCCTGGGCTGGTGACAATTGGGTGAC  CAGAAGTCACAAAGGACTACCCACTAATGGAAGATGGAAAGTGCCCTATG  GTAGGCTCCAAGTCCTGCAGGGAGGTTGCTGAAGCCATCTTATTTGGGGG  ACACTTTGAGATGGTGAGAGGAGGGGAGTAATAGTGCCTTTGCTGTCCGA  CCTCGTCCCTCATCCCAGTGGGGGCGGGGCGGGTGTTGTGAGGATCGCTA  TTCACTAATTACACCTTGGTGAGCACCAAGCTCCTTGGAGGGCAGAGAAG  CCACATCAATACAAAATAGCAACCAAACAGTGCTCCTGATGCAGCCTAAT  GACTCTTCTCTGGGCCTCCAGACCTGTGCTTCTGTGCATCACAGACCACA  TGGTTCCATCTCAACTTATGCTGGTGCCATCACCTGGTTCCTTCACAAAG  CCTCCCCAGCATCCACCCCTGATTGCTAGAAAAACTACTGCCCCTCTCAA  CAGATCTTTTAACCACAGCAGTTATTCCTGTCTTCAGTTTTAAACCTACC  CTCAGTCACCTCTGCTCCTTTGAGACGAGGGACAATGGATTTGCCAGCAT  CCGGCATCTTTTCAGTGGGGGGAATGAGAGTGTATCCTCCACCGTGATTG  TTCTGCCAGGATGGGGTAGGGGAAGAGAAATGGGAGGTAATGGGTCCTTT  TCCTGTCCTCCCCGATCCTTCCAAGGCTTCAAGCCCCACGTCCTGGCCTT  ACTTTTAACAACGGCAGGGGAGCCCACAGAGAAAGGCAGTCACTTTCACG  AGACAGTGTTTGCGAACGTAAACGGCCTCCCTGTGTGGATTTAGGAGGTT  TCAGAGGAGCCTGACAGGCAGCTTGCACAGTGCCAAGCTAACTACTTCTG  AAAGGGGGCCATATGCTGCCCTTACAGCACCCCTTTGGTGCTTAATTTGA  TGAAACAATGCACCGTAATCAGCCATGAGGACAGCAGCCGTGGTAGATGG  AGGGCGACTCACGTGGTCCAGCCCCGAGTTCACCATGCTAATCCCATTAC  TCGCCTGAGTTGGCTTGTTTTGGGCTACCTTAAAAAAAAGTTATTTCTCT  ATTGGTCAAACTGCCGTGAGGTCTGGAAGATTTAATGTATCATAAGACTC  ATTAAATAACCAGCCTGGCAGCACCCACTGCTGGGCAGAATGCTGGCATA  TGGAGGAATCCTGGCCGAAGAATTGATATTCACTCACACAAACACCACTC  AGCTGAGGCCTCAAGGGACAAGGCTGGTGTGTCCGGAGAAGTGTAAAGTG  TGTTGGAAGCCAGTGTCGCTCACTCTTTAATGGATACGTAAGTCAAGAGA  CCTTACTAAAATTCAAATTCTGGTTCCTTCAGTCTAGGGAGTGGGGGCAC  AGACCCGATATGTTGCTAACCAGGTGACACTGAGGCCACTGGTCTATACA  AGTTTCACATTTGAGATAGGCGAGCACTAGACAAACCTGAGAGCAGCCAA  TAACAGCATCTAACACTTCTTAAGTAGGAACAAACCACTATGTTGTCGAA |
| 90,992,944 | 90,994,943 | CTTCCGGTGCCCCGTATTCTGTGGCTCGGATGGCATCGTTAAAAAAACAA  GCCAGGTCATATTGGGGATGAGGGGCCGTTCCTTTCCTACAGCCCAAGGT  GCTTGCTTTACTAATAGCCATTGGCTTTGCAAGTTACTGTTGGTCTCTCC  GAGCTGCAAAATGCCAGCGCCGGGGCCGGTCACAGACAGGGAAAAAGTTG  GAAGGACTTTTTAGTTAATTCACAATTGCTGCTTGCAGAGTTCCCCAAAG  TTGGGGTGTGGGAATGTGTTTGGGGCGCAGTGCAAAATTTAACAATGGAC  TTAATGACGTGTGAGAAAGAAAAGCCTAGGATGACAAGGAAAGTGGCCAT  CCTGGCCAAGTCCGTGTGGCAACAAAGAACGGAATTCTTAAGGTAGAGAC  TTAGATCCGCCTTGAAGGCAATCAAACTTGTAACCATCACTCTGCTGGTT  TTCTCTATAAATTAACACAAAGCCAGTTTAATCTAAAAATTATCATGCAG  CAAATAATAAATAAATAAATAAAAATAAAGAAAAAAGAAAAGGAAAGAAA  AAAAATTCACCTTATTTACCACAGTTTTATCCCTCAATGCTTCTGTGTTA  GTTTAATTTGCTCACCAAACTTTTTTTAAAAAGATAAAGTAAACTTTATT  GAGTTTAAGCATATTTGCCTATGAAATGTTTTGGAAATCTAAATGACCCA  CCTTAGACAACTAATGCAATTTAAAAGTAGAGAGACCTTGCTTCCAGGTT  GAAGCAACAGGTATTAAAATGGATAGAAACAGAGTTTTTGACCTCACTCT  CTGCTCTGTTGCTAAAATCTCACATCTCCAATGAGTCCTGTTAGTTTTCA  GCTGCTTTCTGAGGAGCAGGGTGCTGGCAGACAGACTGTTTCCCTGCTAA  ACAATAGTCCTGTTTAATTTAACGCCTTCAGCAAAAACCCAACAATATCC  CACAAGACCATTACACACTAGTTTACCCTTCTCTTTTCGTTTTTTCATGT  TTCACTTTCGGGAGCAGGGAGGGGAGGGGACAAGGCTAAAAACAAACTAA  TTTTTGGTCATTTTATTCCTTTCGCAAACAGTTTGAAGACTAACCAATTT  GTACTTACAAATCTGGGGCATTAGCAGACGGGGACCCTAAATCCGCATGC  ACACAAGCGGTAACTGTTTGTATGCCAAGCTGTTTGTAAGCCTGATTTGA  CAGCAGAGAGCTATTCATTCCTTTGTCAACTGTCAGTCTAAAAACGTTAA  CCTGCCTGGGTCCCGGGGTATGGGAGCGCTAAGTCTTCCCTCCTTCAACT  GTATAATGAACAGACTGTTTCATTAATGAGACAATTCACTCCACAAATCC  AGCCCAAACAACTTATTTCACAATTTGTGAGCAGAATTAAATTTCAGGGG  AGTCGCAAGAGGGGGTGGGAGGGAGGGGGGAGTAGGTGAGTTGTGCACAA  TATATCAAGGGATCAATGCGTTGGGATAAGTGTCCCAGGCAATTTTGCCA  GCGGTTGTGGGCCGAGGCTTGGGAGGCCACGGCATGCAGATCAGCGGATT  AGGTGGGGGGAGCGCAGCATTAGGTCAAGTGTCTCACGGGGAGCTGATAA  ACACCCCAAACCTGACAACAGATTTTCCTTTCTTCCAGCACCACTTGGTA  AGTGTCAGAGCAAATCTTCTGAATGGCAGTGCGCTTTACTTAGAGGCCTT  TCTTCAGGGGGACACTCTGTCCATTTTCCTGAAGCCCATTCCAGGCTGCT  TCAGGAATAAATTTGCCCGGCATCTCACACTCACTCGCCACACACACACA  CACACACACACACACACACACACACACACACCACACCGCCTTTCCCTCCA  TATCTTAACTATAAGCCTTTGTCTCAAGGTGTGTGAACCAGCCCCAAAAT  CCACATCGAGAGTCCCTGAGGTTTCAGAGGCCCTGGATTTAGCTTTAATA  CAAATTAAGCACTTTGAAGATTATACAATAAAAAAACCTCCTCCCTCCCT |
| 91,002,382 | 91,004,381 | GCTGGGGACCCTCCTGTGTCAGCCTCTTATCCTCCTAGAGGAGTGCGCTG  GGATCACAGAACTTGCAAGTGTTTCATCTGCAGTTTTATGGGGGCTCCCG  GGCAGGGCACAAACGAAGCTTGTCCAGTCATTCTTTTTTTTTTTTTTTTA  TAGTTTAATGTATTTTAATAGCAAACTTACAGGAACAGCACAGAAGACTG  ACAACATTAAAAACATGTACTTGTATGTAGGACAACTCAGTTAGAAAAGT  ATAGTGAATGGATGGAATCTACTGTGTGATAAAAATGCTACAAACACCAT  TTAGTTGCCGTCAATAAGAAATTTACTTATTTTTAAAAAATCCAAATGCT  GGCATTGTCCAGAAAATTTTAACAGGTTTATTTATAATTGTTATAAAGTT  GAACTGTTGAAATGTGTTCACTGAAACATTTTGCTTGCATTAATGCTTTC  CATCTTGCATTTATATTAAAAATTCACACACAAATGAACGTGGAGAAACG  CCAATACCTGATTCTGTCCCCTATGTTTCCACTCGCAATCATATACTTAG  GTACCTTTGACCCCATGGAAAAAATATCTAACATTCAGAACTACTGATAA  CAGGAAGAAGAGGAAAAAAAAATTTTTTTTTTTTGAGAATGAAATGTTTC  CCCTCATAGTGGACTCTTAAGCACGTTCTCCGCGTATGCGGCATGCTAGC  TGGATATCTTTTGGCATAATTGTTACACGTTTGGCATGGACAGCACACAG  ATTGGTATCTTCAAAAAGGCCAACCTGGTAGGCCTCACTTGCCTCCTGCA  AAGCACCAATAGCTGCACTCTGGAAGCGCAGATCTGTTTTGAAGTCCTGA  GCAATTTCTCGCACCAGACGCTGAAAGGGGAGCTTGCGGATCAGAAGTTC  AGTGGACTTCTGATAGCGTCTGATTTCACGGAGTGCCACAGTACCAGGCC  TGTAACGATGAGGTTTCTTCACCCCTCCAGTAGAGGGCGCACTCTTGCGA  GCGGCTTTTGTAGCCAGTTGTTTCCTGGGTGCTTTACCACCGGTGGATTT  GCGGGCAGTCTGCTTTGTACGAGCCATGGTATGGACACCTCCTTACTTAC  CCCCCTTCTCCTTCGGCTGGAGCTCGGCAAGCGAGAGGCGGCACTGGCAT  TGGAGAGCTGCGGCTGCGCGGCTAAGGCTGCCCAGTCATTCTTACATAAC  AAGTGCTTTTTGTCACTGGGCATCTCCCCACCCCAGATTGCGTTTATTCT  TAAAGGCCAAGTGCAGTGAGCTTTGCTGTCTCCCCAGTGCATTCTGTACC  TTACTGATTCTCAATGTAAGTAGTTCACTACTGGCGCAGATATTTCCAAG  TCATGTTTTTCCCCCTACCAGTTCTTTCTTAGTTCACTTAAGAAACAAAC  AAAAACCCATTTGAGTGGGAATCTCTTGAAGCCACAATATGACTTTATGG  CCACTTAACCTCGAGAGGAAAACTCCATGCGCTTCTCCAGCTGGGGTTTG  TAGGATCTGAATGTCTAGCTCTGCAGTATTACCATGGTGAGTGAGGCCAC  ACCAGGACACAGAGGGTGTCAGGGGTCAAATTCACGTGTTTGAGAAGGTG  ACAGAGTTGGGGAGGGGAAGGCCTGTGGCTCTCCGTCCAGCAATGGAAAC  CGTATCCGGCTGGCCCAGCTCCCGAGCAAACTGAAACAATATCTGAAGCG  CACATGCTTCACTTTAGATTTGAAGGGTAATTTAATTTTTAAACACTGCT  CTTACTGAATCATCAGTTTTTAATAAACATTCAAAAGAAGAAAGAAATAA  GAAGAAAGAAAGATGGGAGGAGGAGAGAGGCAGAGAGCACAAGAGCAAGC  TGTCGAAGAAAGGAATCATCTCTCCGCTCAGCGGCTGCCCAAGTGGAGGA  AGAGTTTCTATCGAAAAACTACTCCTGGGAGCCTCACACTTCCTCCTCCC  TCCCTCTTCCCTTTCTTCTCCTTCACTCCCTCTTGCTGGCTTTCTTTTTT |
| 91,028,363 | 91,030,362 | TCCATGGAGAAGAAGTGGGACTGTCCTGACAGCACTCTCTCTGCTCAAAG  AATGGAGGGAGGGGATCCATGTGACCTCACATGAAGCCTCTGCCAGCTGC  AGCTTGGACATGCCAAGTTCTGGAATGCAGCTTCTGGGCAGCGGCAGCTA  GAGCGCTCTGGCTCTGTGCACAGCAGGCCCTGGCTGTCCTGATAAGAACT  ACAGGCCCCGGGAGAACCAGGCGGCTCATCTCCCTGTCAGGGGGCTCTCG  CCAGCCTGGCTTGCTCTACATTGCTGCTCAGCGTTAGTGATGACCCGTCT  GCAGATTTTAATCCGTATAGATTCCCAGAGCCATTGCTTTGAAGACTGGC  GGCTATGCTAGGTCTGTAGGCAGGCAAGGAGGAAGAAGAGACAGGTGGGA  AGGGTGGGGGGCGCTGTCCTTTCCCCTCTCTCTGTAGTCTCCCCCTGAGC  AGAAGCCTTCCCCTCTGCCTCTACAAGGGACAAGGGATGAGAAAAAGGCT  CGCTTACACTCCCAAAGAAGCACAAAGAAAATCAATGTGTCTCCCCGACG  TGCTCGTGTGAGCTCCGCCAATTTCTTCAAGCCCCTGGTGTTCAGCGGGT  CAGCCCAGTTCCCAACCCAGTCCTCAACTGGATTTAGAAGGCGGGGACCC  AGACAGCACTCAAATCCCTCATGTGGAGCCTTGCTGCTTCTCATTTGTAT  AAGCTACAGCGGAGGAAAGTCTCAGAACTTTCCCGGGCTATAGGACTCCG  CTTTGAAGCAGTTCCCAACAGTAATTCTGTAATGGACACAAAGCTTGCTT  ACACCGAGTATCTCCCCTGCAGAACTCACAGCCCGGCTTGCAAGGATGGC  AGTGCGGGACTCCCTCGGGGAATGAGCTACCCTGGCCAGAGATCGGCTGA  TTTTTTTTTTTTTTCTTTCCTGACACAGACCCATAAACACATAGATACCG  AAGAGTTAATTTGCGTTTTTTAAACCCCCTTTCCAATGCCCGCTCTCATT  CCTATAAAATCTCCATGCCCAGACATCAAATCAGTAAGCCCTGTTGCCTG  TCAGTGAATACAATGTCAGGCCATTAGTATGTTAATAAGTCCGGAGGGCG  ACAGGTGGCCTACTAGACAGCTCAATTAAGCTGCTCATTAAACACACGCT  AACGAACAGCCAAGGCAGGGCCTGAACAGCACCATTCTGTAATGACATGA  TGTCAGAAGGAACAAATGAGGAAAAATAACAAAAACATTACAGCCGGGAC  AGACGCCACTAACAAGAACCCCTCGCACTACATCAGCCCCCAAATTAATG  AGTCACAGGGATCAAAAAACTCGACCCAACATTCTCAGGAGGCTGAATGG  AGCAAGATTTTTTTTTTTTTTTTTTTGGTCACTATTAAAAAAAAAAAATC  TCCAAACCTAAAATGACTGCAAAAAAAAAAAAAAAATTTAACTGCCGTCA  ATTATTAAAGCAAAGTTTATTTGACACGACTAATAAGGAGGTCTGAGAAA  TTCGTATTATGGAATTTGAATGAAAATCATTATTTAATGAGATCATCATT  GACATTTGATTGGCTTTCTTTCCCCCTTGCACAGGTTTGAGCCGTTTGGC  TTCTGTACTGCCCGTCCAGTCTGAGCGCACAGCAAGCGAGCCGAATGAGA  AGGAGCCCCCGGGCCTGCTGCTCAGGTGAGGCTCCTACTTGTTAGGGATG  TAAGCCCCGGGACTTCCAGCTGCTGCTAGGCGTTATCTCATTTCCTAAGA  AGTAGCTTCCAGAAGCAAACCTCTGCGGGGACCACTGCTGACCTTGGAGA  GACGCACTGCTAAACTAACAGGCTGCGGGGCCCTGACCACAGCTCTGAAG  CTGACACCCACCTTGCAGTGAGGACTTTGCTCCAGGTTCTAGGGCTGCGC  GGGGCGGAAGCTGGGAGAAGGCAGGGGACCACCCACTCTGGGTGTGTGTC  TGGGGAATCTTAGCGCATCGCTTGGAAGCACGGCTCTGCCCATGCACTGC |
| 91,051,171 | 91,053,170 | CAGGAGGTAGGAGTAGTCATTTCAGCCAGGGAAAACTGAGTCTGTCTTGG  AGAGTGGGAGATGAACATTGATTATTGATTATTCTTTCCCAGAGGCCATT  AGCACTGCTTCTTGGCACTTTGGACTCACTCTGGTGCCAGTTGCAATAAA  TGAAAACCATATTTAACTTAAAAAGTGAAGCTCACAACGGTATCGTATTG  CACAATGGAGCTGCAGTGGGGCAGGGGGGCAGCTACTGTTTCAAGATGAC  TTGAGAATTCAATACAACGTGGGGCTGTGTGGTTACATGCTTTGAAAGGC  ACCTCACAGATGGGTTTCATAATTGAGAAATGTATTTGTTCTTTGTTGCT  GTGTAAAAATAACATGACTCAAATTTTGGAGTTGGATGCCTTCCTGGAAT  GGTTTCCCTCCACCTTACAACACTTAGGGGGCTGCACGGCCACAGCGTCG  GGTGGGTGGTGGGGTTATAAGTGCAAGCCTTTGGACACACCACAGGGAAA  AGCTAAAGGAGTTTGCAAACAGATGCTGAAGAAGACAAGGGATATCTCGA  GCCAGCACCCTGAGGCCTGCGTGGTGTCCAGCAAGGTAGCAAGGGGGAGA  AATGATGGAGTCATTCTTGGAGTCAAGCAAGCAGGGCTTCACGAAGCAGG  GATGTCACAGGGATTATAGCTCAAGGTAGTTCCCTGCTCCGAGGACACTG  GGGGGCAGGGAGCGGGATCCCAGGGACATCCCTGCTTTGACTTTCTAGAA  CATTCTAAGATCTCTAGCTCCCAGTAAAAACAAATAAGGTACTCTTAAAA  GGAGTACTCTGTCCAAGTACCAAAGACTAGGACTTCCTACTGCTCCCAGG  AACAGGACCTTCTCACAGCCCTTGCTCTGAATCTGCCCCTTCAGGGTCAT  TTTTCAACATTAGAACATTGTTCAGCCTTCCCATGTTCCTTGGATTGTGG  AACTAAAGGAATCTTGTGGGTGTCTTAAGTCCTCCAAAGATACATGCTCT  GAGGTTTTATTCTGGGACGGGGAAGACATCAGTTCTGGTTTTCCTGCCCC  TTGAGTAATAACATGCAAACAACCATGTTCCTAACAGACTGTAGGTCATG  GGGCTTGCCAGTGACTTGTGGTGGCTTAGAGAGAGAGCTTCCCCTCACCC  CCTGCCCCCTGCCCCCCCCCCCAGACACTGCCGGTCCAGCGAGCTGCAGC  TGAATGTTCGCAAACTCTAGCCAGGTGCAGACTCAGACTGTAGTAGCTCT  TGCAGAGTTCCTGGTTCCCCAGCACTTGAAGGAGACCTAGAAGATACTTT  CTTACAAGCTCTGCCACCGTGGTGGCCGCGGCACCACCACAGCTGCAGCT  ACTACTGGGATCTCAGAAGTAAGGCTTTGCTTCTGGACTCCGGGAATAAG  GATCAGGAAGATGGAGAGATGTGGTTTAACGGTTCCCAGACACTTCCCAC  CGAAGGTCTTACAACTATTAAGCAAACTTGAAGACTAACTTGGTCCAGGC  TTAGCTATCCCAAGATCAAGACTGGTGTCCAGGGAAGGCTAGTGTCTTGT  GGAAGAACTCTATGCCCAAATGGTCTAAAAGTGGATAGCTGTGTCACCGG  AGACTCGGAAGATAACTCAGTTTCCAGATGGTTTAGCTGAGGGACACCCT  CCAGGAGGTGCCCGGCTTTGTGACAGACATATCAGTGAGTGGGAGCTGGT  GGATCTCTGGGGTCAGGAGCTGATGGGTGATGGGAGATAAGACTGTAACC  AGCCATGTTGGCATGTGTGCCCCCTGCAGGCTGAGCCCTGGGAACATGGG  ATCTGCCCTCCTCTCCCCTCTCCCAGAAGCAGGGTTCTGAATAATACTTA  AGTGACAAATTCATTTAAAAACACAAAACCACACAGGCAGAGCTCTCTGG  TTAAAGTGGGGGGGGCTGTTATGGGAGGAGCCTGGAGGTGGGGGAGGACA  GAGAGACTGGTGTCCTTGACATCTTTCTCACATGGCTTTCTCATGACCTA |
| 91,061,447 | 91,063,446 | TAAGTAAATGCTATTAAACAATTAAACATGCAACGTCTTTCACAGCTGAC  GACTGAAGACTTTGCTCTGTCCGTTAGCGGCAGTTTTAATTAACCACAGT  GTGAGTCTCAAGTGGCGCTGGGCGTATGAAGGATAAATGATCGTGGCTTG  TTGGGAGTCCCCAGTTTGGGTGCCAATGTAACAAGCAGCTAAGAGCATTT  TAGCTTTGACAGGCGTTCAGTGATGGAGTCTTAGCTGAGACCTTTCTCAG  CCTGGATCCTGTACCTAAGCCCAGATGAATGAATTACGATGGAACACAAA  GGGAAAACACCACAGACTAGACGTTTTCCTGGCTGTATTTTAAAAACTGG  ACATTGAAATATGGGATTTAAAAACAAAAACAAACAAACAACGACAACAA  CAACAAAAAACCTCTACAGATAGCTTGGGGACCTTTAGACTTGGAGACTT  GGTTTAAGAAAGTCTTTCGCCTGCCTGTTTAGAGGTGTTTCTATAAAAGT  GTGTGTACATGGATATGAGTGTGCACACACTCTAGTGCAAGCGCATGCAA  GCCTGTGTCTCTGACGATACCCTTAGAGATGGAGCGTTTCTCCTGCCAAA  ATACACACACAGCCCAGGATTAAAGGCGCTGAAACCTACCAGCAAGTTCT  TAACGCCCTCCCACAATCATTAGACCAAATCCACATCATTCTAGATTCAT  TTTCTCCTTTATTCATCCGTCCAACTAATTCCCCCCGCATTCACCAGGCT  TATATCTTAATCTTTTCTGTTACATGGAGAATACATAATGAAAAGCTGCA  TTTTCTGGGTACATGCACACATTGGGAACGGATGAACCATACGTGAAGTG  ACATACAATGCTGAATGCTGGCTGAATGGTCTCCAGCACTAAAAACACGG  CGAACCCAGGAACAAAAAGCAGACGAACAAGAGCCAGCGCCGGCCCGGCC  TCCGCAAGGACACCAGGGGATTCGCCGGGGAAAACATGGGTATAATTTCA  GTGTTTTCAATCACGCAAGCTGTGGGAAAGGAGTCACCATCTGATGTTTG  ATTGTGTGGGATATGTTTTTGGCAGTGGCCCTGTATTCTGCATTGACAAA  AAAAAAAAAAAAAAAAAAGTAAAAGAAAAAAAAAAGAAAGAAAGAGAAAA  GTGGGGGAGGTGGAGGCGGGGCAAGGAAATAAAAAAGAATGCAGTTACAT  TTGCAGTTTTCAATTTGCTGCTTTGGGGACAGACACAGCTGACAGTTCTG  ACCAGTGGCCACCTGAAAATTAGGGCCAGGACCTGGCTACTGTCCCCTCT  GTGGTGTCCTCCCCCGCCCCAACTGCCTGGGCCTCCTTTTATGCTCCATG  CTACCACAGGGGACTTTTATCCACTCTCCCCGTTGCCTGGTTAACTTACA  GCTGAGCTGTGGGGCTGATGTTTGCCTGATTTAATAAAGTCTTTTATCTG  AGGTAGTGGCTGAGGAAGGAAGGTGGGGGACCTGGCAGAGGGGAAGGGGC  CCCGGCTCTCACCAGCAGGCATTTCCTGGCAGGGATGGAGACAAAAGGGG  AGGGTGGCCAGAGGACTCCCAGGCAACATGTGCAGGACTGGCACTCAGCT  GCCAGGTGGCTCCAGAGGGTAAGTGTCCAGATATAGGGGTTGGCCATGTG  GTTTGTGGATGGAGGCAGTCAGTAGTGGTTAGCAGCCACAGATTCCAGAA  AGAGCTAGGTTTGTCCTCACCCAGACTGCTCAATTTAACAAATAAACACT  CGGGATGCCGGGCCAGATCTGTCTCATAAAAACAACAAAACATTAAAACG  ATGAACTTGGAATCCAGATTTGCCCTGTATTTTATCAACCCTGTTTCCAG  GGTTCAAGCGCCCCCTCCTCACCAGCTGGAGAGGGACAGACAACCTCCCC  AATCTGTCTCAGGCCATTCTGAAGATGGTAGGGAAATGGAAGGAATCAGA  GAAAGGGGCCTCTGGGCCTTGCCAGTAAACATGTGCTTCCCACACCCAGC |
| 91,080,626 | 91,082,625 | TCTTCTTTCTGGTCAAGACAAGGTTGTCTCTCTGGGTGGAAAGAGGCCGT  GTGTTCGTGGTCTGCAGCTGCATCTCAAAGGCACTTTGTTGGCTGCTGAT  GAACTTGTCTTTGCTCGTGTGGGGAAACAGGGAGATTCTTTGGCTCCCCC  ACGCACGCTGAAATTGATGTTATTTCTCTGCCAGTGGTACCCAAGGAAAG  GTTTTGTTCCATTTCCTCCCTTTTCAGGCTGAGCCCTATTTATGTTGGGA  GCTGGGGCGCTGCCATGAAGGACCATTGTCGTTTCCCACCAACATCCGAG  GCTGGCCAAATCCAGCAGCTGTGGGTCCATGCCTGCCCTCCCCAAGGCTT  ATTCCCTTGACAACATGGCCTTGTTTGGGGCGACTTAGTCTTTCTCTTGT  GAGCTCCTTCCTTTACTCTCTCCCTCTTCCCAAAGTCACCCTTCCTTCTT  CTCTCTCTCTCTCTCTCTCTCTCTCTCTCTCTCTCTCTCTCTCTCTGTGT  GTGTGTGTGTGTGTGTGTGTGTGTGTGTAGGCCACAAACAGGAGACTGGA  TCTGGGAAGGGGATAAAGACTGGGAGGTGGAGAGAGGGTAGAGTCGACCA  GTGCATGCATGGGGTGAAGGGCGGAAAATTTCACCATAAATAATAGGATC  AAATTCGTTATTATTTAGATGATTGCCAACTGCCTCCAACCCAACTTTGT  AAGCCGAGCCCTCATCCCTACCCACTCCCGGTGTGTCAGATGGAACACCT  AGGTGGCCCACGGGGACCTCTGACCTCTATATCCTCTTCCTTTCTCCCTC  CTTTGCTACTTTTCTACTGGATAAAAGGAGAGAGTGAGAGATAATTAACA  AAAAACATGGCCCCGGGACAATGAAGCAACTGGCCTTGGCCGGCAAGCAA  CGATCCTGGTTTTCTAGGTAGAGTTTCTCCCATCAATCTTTCCTTTAACC  TCCCTGTTCGTGGAAGCAATAGAAACACCACCCCCTCCCCTGAGCAAATG  CTTTCTTTTGACTGGAAACAAAAAGGGGGCCCGGCAAAGACGGAGGTGAA  ATCTGGGTGGTATGGGCACCGCACAATGGCCCCGCTGTTCCTGGCCCTGC  TTGTGTTTTACAACAGGGGAGGGGCAGGCGCGAATGGTCCGATGGTGGAG  ACAATCCCCCTGATTCAGGCTACAAATGCATCTTCTATTCCACACGGAGC  TGAGCAGAAAGGATGGGGGTGACAAAGAGCATGGGCGGGGAGAGGGAAAA  CAAAATGTTTTCAGTTGAAAAAAAAAATCTCTCATATCCTACACATCCTC  AGAAGAGCTTCTATGGAGAAGGCCTTCGGAGAGTCCCAGCCCACAACTCA  AGGGCTTTGTCTGAACTCTGATTTATTGATGAAGCTTAAGCGGCTCGCTA  AGAAAGGCCTGGGGGTGTCTTTGTCTTGAAGATAAAGTACAATAGGCCAC  AAGGGCCAAGATCTCTCGGGATGCTCTCAGGTCCTGCCTCTCTCTTGCCC  TCTCCTCCCTGCAAATGCCAGCAGATGCTGAAAAAAAAAAAAACCCATCG  GTGGTGTGGCTGGGAGTGCTGGGGACAAGCTGGGCCACTTGAGGTCTCCT  TAAGAGGGTATTATGGCCAGGGAAAATATTTGCGCTCTAAGGATGGCACA  CTCCATTTGATAATGGCTCTCATCTGCCTCAGATAATCGCCTCCCTCCCG  GCTGTCAGGGGTGCAGCCACTGCCAATTCACAGCGCCCTCCGAGAAAGTA  CCCTTGTCTGTGATGACCAAGATGGGGACATTGTGTTTACCTACTTGAGC  AGAGGAGAAGGTGACCGTGAGGGCAGCCTGCATTGTAAATTACAATTAAA  ACAGAAACAGACAGTTCCTGCTCTGCCCTGGGACCCCCACCAATAAATTA  TGGGTGGACATTAGGGGAGAGCCCAGGAAAGGTTGGGTCCTGGGGAGGAT  CCCCCCATCCCATAGCCTACCGACAGGTCTTGGATATAGGGATAGGGCTA |
| 91,097,264 | 91,099,263 | CGTTCTTGTTTTTTTTGTTTGTTTTTTTTCCAGAATCAGGAGAGATAATA  ACGGATTTGGGATTTAAGCCCTACATCTTGGGCTGCTCGGTCATGAGCGT  AGATCCCCACCACTTAGGACATAATAGTTAAGTTCTGAGCTTGGCCAACA  AGGCGTGAGCTCCCACTTCCAAGACATGCTTGGATCTCCTTCCAGACTAG  CTTAAGAAAAGTGACTTTCTCTCAAGCACACAGCTGTGGCGCTGCTCACT  AGAACATACATACATGATTGTTTGTTTGTTTTTCAGAAATTTACCAAATC  TTGTAAAACATAACACTAATGAGAACTAAAATGTGTGGGATCTATGCCTA  CATTCAGCTATTCCCTTGCTTGCCAGGAGCATGAAATAAAGGATATGCTA  TAATAAACTTTCCAGTTGAGCTGCCATCACTTCTCGTCAAAATCGGAAGC  AATGATAAGGTCAGAGCCATCTTGGTTAGCCTCCTTTCCCCAAAGCCCCG  CCTCTTATAGAGCCACATTAGGGGGTCAACCTTGCACGATCTAGGCCAGG  GCTGTACATTGGAGCATCCGGGTCTTTCAGAGGAACTATCCGGTATGTAT  GGAAGGCATATGTTTGCAGCGCTCCATAGAAAGACATCATTACTGGGCTC  AGGTTGCCCGCGTCCCTCTCTGGATAATGAGTAAGCTATTTAGAATCTCG  AACTGTTTATGTGACATTTCAATAAGTAATGCAGAGATTCAACAACATTT  CCATATTCCTCTTTTAAAGCCTGATAGGCCCCTGCCTTAATCCAAGCTCT  TTGGCATTTATTACTGTTTGTTACACTAACAAGTAAGATTATGGGAATCC  TAATGAAGGCTTCCAGTGAAAAAGTCCGTGATTAAAATGCCATAATAACA  GTTGCGTGCATTTATGTGCCTGATTACCATAATCTATTTAGCACAAGTAA  ACACACAAACAGAAGGAAGAAGCCCACTGACACTTCGGCTAAGGGGGAAT  TTTAAATGCGAGGGAGACAATGTAAATATGTAAATGTCTTTGTGTATTCT  TTTCATCTCTCTCCTTTCTCATCCTGCTTTCTATTTTTTCCCCCTTCTTT  TCTGTTTGCTAATGAGGAGACATGCCATCCCATGTGGTCCCTCCCTGCTC  CATCAGGCTGAGGTACATTTTGGGGCCATCTGCTGGGGCCAGCATCCTAA  ACAATGCCACCCAGAAAGTGCTGGATATATTTGCAGCCACCTTTGACACC  GTGGAATAGAAGAAATGAGATCTCAGATGATGGTCTTTGATGTGTTCGCT  CTGAACACGTAATTCAGCTGCTACTCCATTAATACAAGGCCCAGACAAGC  ACGGCCATCCTGTGCCTTGGCTCCCTCAATCCCTCCCCCCGCAAACACCA  CATTGTCAACAAATTAAAAGCGATCATTCCAGGTTTCCAAATTAGCCTCC  CTCCGTATCTCTGCCGGCTTCGTTATTTTTAATTCTTCATAAAATGCCAG  GCTGGAGGCAAGATGCCTTCCCAATCCGGCGGGGGATTGCAGGAAAACTT  TCATGAAAAGTGAAATTGAATGTTTTGGTATAAAACCAGGGTAATGGAGT  GGGGGGGGGGGTAGGGTGGGGTGGGGGCAGCCCTACTTGGGCAATAACTC  CAAGAAATTTGCTAGAATCCCAAGTGCATTGCAATAAGGAAACAGCTGTG  GCACGTTTATGTTTTACAGGAGGAAATAATTTTTTCAAAACATGAGCAGG  CAGGTAGCTGGCTTCACCCAAATGAAGAGAAAACATACTCTGAAGGGGAA  AAAAATAGGATTAATTGGATTAATAGAGAAAAACTAAACTTTTGCAGTCA  GTACGGCTGAGACCTGAAGATAATCACACTTAACAATAATTTATAAATAT  ACCTTTGCTTTTATGTCCCCGCAGCTATTTTAATTAGATTTTGTCTTGCG  TGTGTCTGCACAAGTTTACATGTCTTTACAGATGGGCAGACATGTGCACG |
| 91,112,409 | 91,114,408 | TCCCGCATATTGTACCGCCTGAGGTTTGTTAGGGACTTTGGAGATCCACA  TCCCAGCTGGTGGCCCCACAGAGCTCACCCCCACGTACGCCCTCTCTGCG  GTTTGCCAACGAAGGCCCAAGAGTGGTCCTCACATGCCCAAGCTCCTAAT  CCCCACCACAAGCCCCGTGTGACAGATTCTGCTAATTGAACGGTTATGGG  TCATCCTCATAACCGTTGGGCGACATTATCACCACGCTTCAATTCTTCAT  GTTTTAAATACATATTTAACGGATGGCTGCAGAGCCAGCTGGGAAACACG  CGGGTCGGAAAACAATGCTTCAGAAGGCACGAGATGAGGGGCAAACGCGC  CAGCCCACTGACACTCGAGGGTGGACCCCAGGGGGGGGGGCGGGGGAGAA  CTTAGCACGAAATGAGGAGCAATCGCCTGAGTTTTCATGCCTGTTCTGAC  TTTGCTACAAGAAGACCAATTACTGTTTGTCTGCTTGCTTGTTAAAACTA  CATATGCGAATTCAAATACCAAGCAATGAGAAGTAACGAGAAAAGACCGT  TAAAACTCCGTGAGAAGGAATTGGCGCTTATGTTTGGGGGGTGGAGGAGA  GGTAGCACCCGAGATCTGTAAAAAATAAATATCCCAACAAACAAACAAAA  CGCATTTGAGCTGGAAATGTCCGGATGTGAGAAACAGTTGGGGGTGGGGA  TACAACCTACTGCCCCCATGCTTGGGCACTGCCAATTGCTCGCATCCATC  CACGGTAGGCAGGGTCACCTGTATGGGCCTGGCCGTATTTTCTCCTGGAG  GCTTGCTGAAATCAATTCCTAGGTTTTCACTTCCATAAAATGGGTGAGTG  TATAGTTATCTCTAGACTTCCCCAGGCAATTCACCCAGATCCAGTTTCTC  AGCTTTGTATGTGTGTGTGTGTGTGTGTGTGTGTGTGTGTGTGTGTGAGA  GAGAGAGAGAGAGAGAGAGAGAGAGAGAGAGAGAGAGAGAGGCTGCTACC  TATAAAAGACGTCCCAAGGCAGCTAGGAGTGTGTTTCAAGTATCAACTCC  CATCCACAGCGGAGAGCCTCCGATAATTCTTTGCAAATAATGTTGCCTCT  ACTCCAAGGCTTGGGCACCAAGATGTTGGAAGTTCAGTGGCTGGGTTTCC  CTGAAAATGAATGAGGTGGAAGTCGTTAACCATCGATGCGTTGGCGCTTT  GACTGTATTGAAAATAGCTCTCCGGAGCTTCTACACAATCTAAACTTTAT  AATGAATTGCGATTTCTTCCCAGAGTGCTAAGAACCTTTCTCTGCTTCCT  TGCCCGCCCCGTGCTCTCCAAGGCCATGCCCCCACCTGGGGGCGCTACCG  CCTGTCCCCAAAGCTGCAGCAACTGCCACTAAGTAGAAAGGAGCTAAGGG  AGTGGGGGCCTTCTGCGCCGCTAGTGTTTTCTTATGCAACCACAAATGGT  CACAGTTTGATGAAAATCACTCGATAGGTCGGCAGCGGGAAACAGGAGGA  GGCGGGAGGGCTAGCAAAAACTCCAGGTGTGGCAAGCAACCTCGGTGTTC  ATTCTCAGCCATAGAACTGGTTGGCCATCGGACGAGTTTGCAGATTCCCC  GTCTCACTCCGTACCCTGGCCTGACGCTACAGTGACAATATTTATATTGA  AAGTTGCAGGCGTTCCCTCTCTGCCAGGGTCCCCAGACTTCTTTAGCAGC  CCCGCTAGCCCACCCGCTTCTTCAGCTCGCCCTTTCCTCTCCCTGCCCAG  GTCCCTCCTCCCATCCCTCCTCGCGAATCTCTCTACCGCGGGCGCCCATT  GGCTGTGTGCTGAGAGGAGGCGTGTGCCGGGCCCGGCGGGTTTCATTGAG  CGGAATTAGCCGGGTGACATCAGCTTCGCAGCCCCTCGGGCCGGCCCAGC  TCATTGGCGCCTCTGCCCCTCCAGGAAAAGCACATTGTTCCCGCCCCCCG  CCCGGACCACCGCCACCGCCGTCGCTGCTGCCTCCACCCAGCTATAAAAA |
